# Serum microRNA-146a-5p and microRNA-221-3p as potential clinical biomarkers for papillary thyroid carcinoma

**DOI:** 10.1007/s40618-024-02467-3

**Published:** 2024-09-19

**Authors:** Antonella Verrienti, Valeria Pecce, Giorgio Grani, Valeria Del Gatto, Samuele Barp, Marianna Maranghi, Laura Giacomelli, Cira Di Gioia, Marco Biffoni, Sebastiano Filetti, Cosimo Durante, Marialuisa Sponziello

**Affiliations:** 1 Department of Translational and Precision Medicine, Sapienza University of Rome, V.le del Policlinico 155, Rome, 00161 Italy; 2 Department of Surgical Sciences, Sapienza University of Rome, Rome, 00161 Italy; 3 Department of Radiological, Oncological and Pathological Sciences, “Sapienza” University of Rome, Rome, 00161 Italy

**Keywords:** Liquid biopsy, Biomarkers, Circulating miRNA, Papillary thyroid cancer, Patient follow-up

## Abstract

**Purpose:**

Papillary thyroid carcinoma (PTC) is the most common malignant thyroid neoplasm, accounting for approximately 85% of all follicular cell-derived thyroid nodules. This study aimed to assess the diagnostic potential of circulating microRNA-146a-5p and microRNA-221-3p as biomarkers for PTC and their usefulness in monitoring disease progression during patient follow-up.

**Methods:**

An observational study was conducted on two cohorts of PTC patients and healthy controls (HCs) using digital PCR. We collected patients’ clinical, biochemical, and imaging data during the post-surgery surveillance. We analyzed the levels of circulating miRNAs in serum samples of patients before surgery and during the follow-up, including those with indeterminate/biochemical incomplete response (IndR/BIR) and residual thyroid tissues (Thy Residue).

**Results:**

Both miR-146a-5p and miR-221-3p were confirmed as effective biomarkers for PTC diagnosis. They enabled differentiation between pre-surgery PTC patients and HCs with an area under the curve (AUC) of 92% and 87.3%, respectively, using a threshold level of 768,545 copies/uL for miR-146a-5p and 389,331 copies/uL for miR-221-3p. It was found that miRNA fold change levels, rather than absolute levels, can be useful during patient follow-up. In particular, we found that a fold change of 2 for miR-146a-5p and 2.2 for miR-221-3p can identify a progressive disease, regardless of the presence of TgAbs or remnant thyroid.

**Conclusion:**

MiRNA-146a-5p and miRNA-221-3p, particularly the former, could be valuable diagnostic biomarkers for PTCs. They also seem to be effective in monitoring disease progression during patient follow-up by evaluating their fold change, even when thyroglobulin is uninformative.

**Supplementary Information:**

The online version contains supplementary material available at 10.1007/s40618-024-02467-3.

## Introduction

Thyroid nodules are prevalent in the general population, detected in up to 50–67% of autopsy studies [1]. While the vast majority of these nodules are benign, only 5% are reported to be malignant. The challenge lies in accurately managing clinically significant nodules while avoiding unnecessary surgeries [2]. In this context, ultrasound (US) examination of thyroid nodules, followed by fine-needle aspiration biopsies for cytological evaluation, remains the gold standard for pre-surgical diagnosis [1, 3]. However, a subset of thyroid nodules presents with indeterminate cytology, necessitating further investigation [4].

In recent decades, molecular testing has emerged as a valuable ancillary tool for risk stratification in cytologically indeterminate thyroid nodules (Bethesda III/IV nodules). These tests help rule out malignancy and refine preoperative cancer risk assessment [2, 5–7]. However, challenges persist when a RAS-like mutation is detected, leaving uncertainty regarding the nodule’s malignancy risk. Consequently, the search for additional diagnostic biomarkers becomes imperative [8].

Among malignant nodules, papillary thyroid carcinoma (PTC) stands out as the most common diagnosis, accounting for approximately 85% of all follicular cell-derived thyroid neoplasms [9]. While most PTC patients have an excellent prognosis, with a 10-year survival rate of 90%, a significant subset (up to 30%) experiences persistent or relapsing disease within a decade after the initial treatment [10]. Therefore, long-term surveillance is required for all patients, albeit with varying follow-up intensities [11], to detect the persistence/recurrence of disease. The early detection of disease progression remains a critical challenge in post-surgery management. Serum thyroglobulin (Tg) serves as the primary marker for follow-up in differentiated thyroid cancers (DTCs) [12]. However, in some circumstances it is unreliable. Indeed, Tg assays could be influenced by the presence of anti-Tg antibodies (TgAb), and Tg usefulness is reduced when normal thyroid remnants are present [13, 14]. The use of radiological techniques also has some limitations: false positive US findings are common; repeated PET/CTs or CT scans increase the burden of ionizing radiation to patients, and thus the risk of second malignancies [15]. Moreover, seriate radiological imaging may fail to detect small dimensional tumor changes, delaying the identification of disease progression.

In recent years, liquid biopsy has emerged as a promising minimally invasive approach. Several studies have investigated the role of circulating microRNAs (miRNA), both free and encapsulated in extracellular vesicles, as potential biomarkers for diagnosis [16–19] and follow-up of thyroid cancer patients [10, 20, 21]. However, results have been inconsistent, and replication across studies remains challenging [22].

Our previous work identified serum miR-146a-5p and miR-221-3p as potential diagnostic markers for PTC [23]. In the present study, we validated these findings using a different technology (digital PCR, dPCR) in larger PTCs and healthy controls (HCs) cohorts. Additionally, we explored the usefulness of both serum miR-146a-5p and miR-221-3p in detecting progressive disease during the patients’ follow-up. By establishing miRNA fold-change cutoffs, we aimed to discriminate patients with cured or stable disease from those with progressive disease. Furthermore, we assessed miRNA levels in patients with indeterminate/biochemical incomplete response, or residual thyroid tissues.

## Materials and methods

### Study design and patient cohorts

We conducted an observational study involving two distinct cohorts of papillary thyroid cancer patients.

#### Cohort I—Diagnostic study

##### Prospective design

Cohort I comprised 72 consecutive PTC patients undergoing care at Sapienza University of Rome between 2012 and 2017. We collected one pre-surgery sample (Pre-PTC) from each patient. Pre-PTC sera from 23 out of 72 patients were also analyzed in our previous study [23]. Table 1 and Table S1 show the clinicopathological features of these patients.

**Table 1:** Clinicopathological features of HCs and PTCs with pre-surgery blood samples

	HCs (*n* = 45)	PTC Pre-surgery (*n* = 72)	*p*-value	miR-146a-5p *p*-value^¶^	miR-221-3p *p*-value^¶^
**miR-146a-5p copies/mL**,** median (range)**	516,138 (29,890-2,235,669)	1,562,719 (273,541 − 15,872,191)	< 0,0001^†^		
**miR-221-3p copies/mL**,** median (range)**	162,540 (3,928-2,231,293)	796,224 (75,108 − 14,422,715)	< 0,0001^†^		
**Sex**	**N (%)**	**N (%)**	ns^§^	ns^†^	ns^†^
F (%)	23 (51.1)	49 (68.1)			
M (%)	22 (48.9)	23 (31.9)			
**Age at diagnosis (median**,** range)**	43 (23–76)	46 (16–77)	ns^†^		
**PTC histological variant**		**N (%)**		ns^†^	ns^†^
Classical (%)		52 (72.2)			
Follicular (%)		16 (22.2)			
other (%)		4 (5.6)			
**ATA Risk**		**N (%)**		ns^†^	ns^†^
Low (%)		31 (43.1)			
Intermediate (%)		40 (55.6)			
NA (%)		1 (1.3)			
**Autoimmunity**		**N (%)**		ns^†^	ns^†^
Yes (%)		21 (29.1)			
No (%)		49 (68.1)			
NA (%)		2 (2.8)			
**Tumor**		**N (%)**		ns^‡^	ns^‡^
T1 (%)		63 (87.5)			
T2 (%)		5 (6.9)			
T3 (%)		3 (4.1)			
T4 (%)		0 (0)			
NA (%)		1 (1.3)			
**Lymph node metastasis**		**N (%)**		ns^†^	ns^†^
N0/Nx (%)		44 (61.1)			
N1 (%)		27 (37.5)			
NA (%)		1 (1.3)			
**Distant metastasis**		**N (%)**		na	na
M0/Mx (%)		69 (95.8)			
M1 (%)		2 (2.8)			
NA (%)		1 (1.3)			
**Tumor foci**		**N (%)**		ns^‡^	ns^‡^
Unifocality (%)		47 (65.3)			
sx (%)		22 (46.8)			
dx (%)		19 (40.4)			
Isthmus (%)		5 (10.7)			
NA (%)		1 (2.1)			
Multifocality (%)		24 (33.4)			
unilateral		6 (25)			
bilateral		18 (75)			
NA (%)		1 (1.3)			
**Extrathyroidal extension**		**N (%)**		ns^†^	ns^†^
No (%)		41 (57)			
Yes (microscopic) (%)		30 (41.7)			
NA (%)		1 (1.3)			

na, not assessed; ^†^ Mann-Whitney test; ^‡^Kruskal-Wallis test with Dunn’s multiple comparisons; ^§^ Fisher’s exact test; ^¶^ p-value refers to the PTC group

#### Cohort II—follow-up study

##### Retrospective design

Cohort II consisted of 50 PTC patients who met the specific selection criteria reported below. Thirty-five of these patients overlapped with cohort I. Table 2 shows the clinicopathological features of these patients.

**Table 2:** Clinicopathological features of PTC patients with post-surgery blood samples (*N* = 50)

**Sex**	**N** **(%)**
F (%)	31 (62)
M (%)	19 (38)
**Age at diagnosis (median, range)**	51 (12–76)
**PTC histological variant**	**N (%)**
Classical (%)	34 (68)
Follicular (%)	10 (20)
Tall cell (%)	2 (4)
other (%)	4 (8)
**Autoimmunity**	**N (%)**
Yes	13 (26)
No	37 (74)
**Tumor**	**N (%)**
T1 (%)	39 (78)
T2 (%)	3 (6)
T3 (%)	8 (16)
T4 (%)	0 (0)
**Lymph node metastasis**	**N (%)**
N0/Nx (%)	30 (60)
N1 (%)	20 (40)
**Distant metastasis**	**N (%)**
M0/Mx (%)	42 (84)
M1 (%)	8 (16)
**Tumor foci**	**N (%)**
Unifocality (%)	24 (48)
sx (%)	13 (54.1)
dx (%)	10 (41.7)
isthmus (%)	1 (4.2)
Multifocality (%)	26 (52)
**Extrathyroidal extension**	**N (%)**
No (%)	29 (58)
Yes (microscopic) (%)	21 (42)
**ATA Risk**	**N (%)**
Low (%)	25 (50)
Intermediate (%)	17 (34)
High	8 (16)
**RRA**	**N (%)**
Yes	14 (28)
No	36 (72)
**“Interim” response at the first post-surgery sample analyzed (I)**	**N (%)**
median (min-max) ^†^	3 (1-172)
ER	12 (24)
IndR	22 (44)
BIR	6 (12)
SIR	10 (20)
**Disease status at the second post-surgery sample analyzed (II)**	**N (%)**
median (min-max) ^†^	24 (11–198)
ER	15 (30)
IndR	16 (32)
BIR	6 (12)
SIR	13 (26)
**Disease status at the last follow-up visit**	**N (%)**
median (min-max) ^‡^	8 (2–17)
ER	21 (42)
IndR	14 (28)
BIR	2 (4)
SIR	13 26)

^†^Months; ^‡^ years; ER, excellent response; IndR, indeterminate response; BIR, biochemical incomplete response; SIR, structural incomplete response

All enrolled PTC patients had no evidence of other primary malignancies.

For all tumors, the final diagnosis of PTC was based on pathological examination. The tumors were staged according to the American Joint Committee on Cancer Staging Manual, 8th edition [24], and the risk of disease recurrence (i.e., high, intermediate, or low) was estimated according to the 2015 ATA risk stratification system [12]. We collected clinical, biochemical (TSH, Tg, and TgAb), and imaging data of patients during the post-treatment surveillance. The outcomes at each follow-up visit have been classified according to the response-to-therapy criteria recommended by the ESMO Guidelines [25]. For this study, we considered three response categories: excellent (ER), indeterminate and biochemical incomplete (IndR/BIR), and structural incomplete (SIR). The RECIST (version 1.1) criteria were used to define progressive disease [26]. Forty-five age- and sex-matched healthy controls (HCs) with no evidence of thyroid disease based on the results of a screening examination (neck ultrasound findings, and results of thyroid hormone and thyroid antibody assays), and with no family histories of thyroid diseases have been enrolled.

The local ethics committee approved the study protocol, and written informed consent was obtained from all patients whose serum samples were analyzed.

### Blood collection

Blood samples (8 to 10 mL) were collected in a BD Vacutainer^®^ Tube (Becton, Dickinson and Company, Melbourne, Australia). The serum obtained after a centrifugation at 3000 rpm for 10 min was stored at − 80 °C before analysis.

Preoperative blood samples were collected from cohort I on the day of thyroidectomy, before anesthesia.

In cohort II, two post-surgery blood samples were collected at 1–3 months (I) and 1–5 years (II) after thyroidectomy for patients without structural evidence of disease. For those with structural incomplete response (SIR), the samples were selected according to these selection criteria: (1) taking the first available sample at least one year after radioactive iodine (RAI) treatment, and (2) ensuring no local or systemic treatments were administered between the two blood samples. Thus, sample I was collected at 1–14 years and sample II at 2–16 years.

### RNA isolation from serum

Total RNA was extracted from 200 µL of serum after the addition of 2 µL of cel-miR-39 (4 × 10^9^ copies/ µL) as the spiked-in control (Qiagen, Hilden, Germany), using the miRNeasy Serum/Plasma kit following the manufacturer’s instructions. RNA was quantified using Qubit fluorescence-based assays for miRNA (Qubit^®^, Thermo Fisher Scientific).

### Analysis of circulating miRNA

We reverse transcribed 0.5 ng of RNA by performing three singleplex reactions with specific miRNA primers included in the TaqMan MicroRNA assays (hsa-miR-146a-5p assay ID000468, hsa-miR-221-3p assay ID000524, and cel-miR-39 assay ID000200) (Thermo Fisher Scientific, Inc.), using the High-Capacity cDNA Reverse Transcription Kit (Thermo Fisher Scientific, Inc.). For each reaction, we used 0.075 µl dNTPs, 0.75 µl RT Buffer, 1.5 µl of primer, 0.095 µl of RNase inhibitor, 0.5 µl of MultiScribe enzyme, and DNase/RNase free H_2_O up to the final volume of 7.5 µl. The absolute miRNA levels have been quantified on a chip-based platform using the QuantStudio 3D Digital PCR System (Thermo Fisher Scientific, Inc.). For each sample, we performed two duplex assay dPCR reactions to quantify one target miRNA and the spike-in control simultaneously. For each reaction, we used 1.5 µl of target and control miRNA RT reactions, 0.8 µl of target and control TaqMan MicroRNA assays (with FAM™ and VIC™ dye-labeled MGB probes, respectively), 8 µl of Master Mix and DNase/RNase free H2O up to a final volume of 16 µl. We loaded on the chip 15 µl of the reaction, following the manufacturer’s instructions. Target miRNA abundance expressed as copies/µl has been normalized as follows: n. copies/µl of each target miRNA multiplied by a normalization factor (NF), where NF= [average of spike-in (copies/µl) of the two chips for each sample]/ [median of all spike-in (copies/µl) of all chips].

The final absolute miRNA levels have been expressed as copies/mL.

### Statistical analysis

All analyses were performed with GraphPad Prism software, version 5.0 (GraphPad Software Inc., San Diego, CA). The Mann-Whitney test was used to assess differences in miRNA expression levels between the two groups. The Kruskal-Wallis test followed by Dunn’s multiple comparisons test was used when more than 2 groups were compared. Fisher exact test was used to analyze categorical data. P values lower than 0.05 were considered statistically significant. Receiver-operating characteristic (ROC) curves and areas under the ROC curve were analyzed for both miRNAs.

## Results

### Evaluation of miR-146a-5p and mir-221-3p as potential diagnostic markers for PTCs

Pre-surgery sera from 72 PTC patients and 45 age and sex-matched healthy controls (HCs) were analyzed for circulating levels of miR-146a-5p and miR-221-3p.

The clinicopathological features of PTCs are reported in Table 1. No difference in miRNA levels has been observed based on the clinicopathological data (Table 1, Table S1). Notably, both miR-146a-5p and miR-221-3p levels were significantly higher in pre-surgery sera of PTC patients compared to HCs (*p* < 0.0001).

**Fig. 1:**
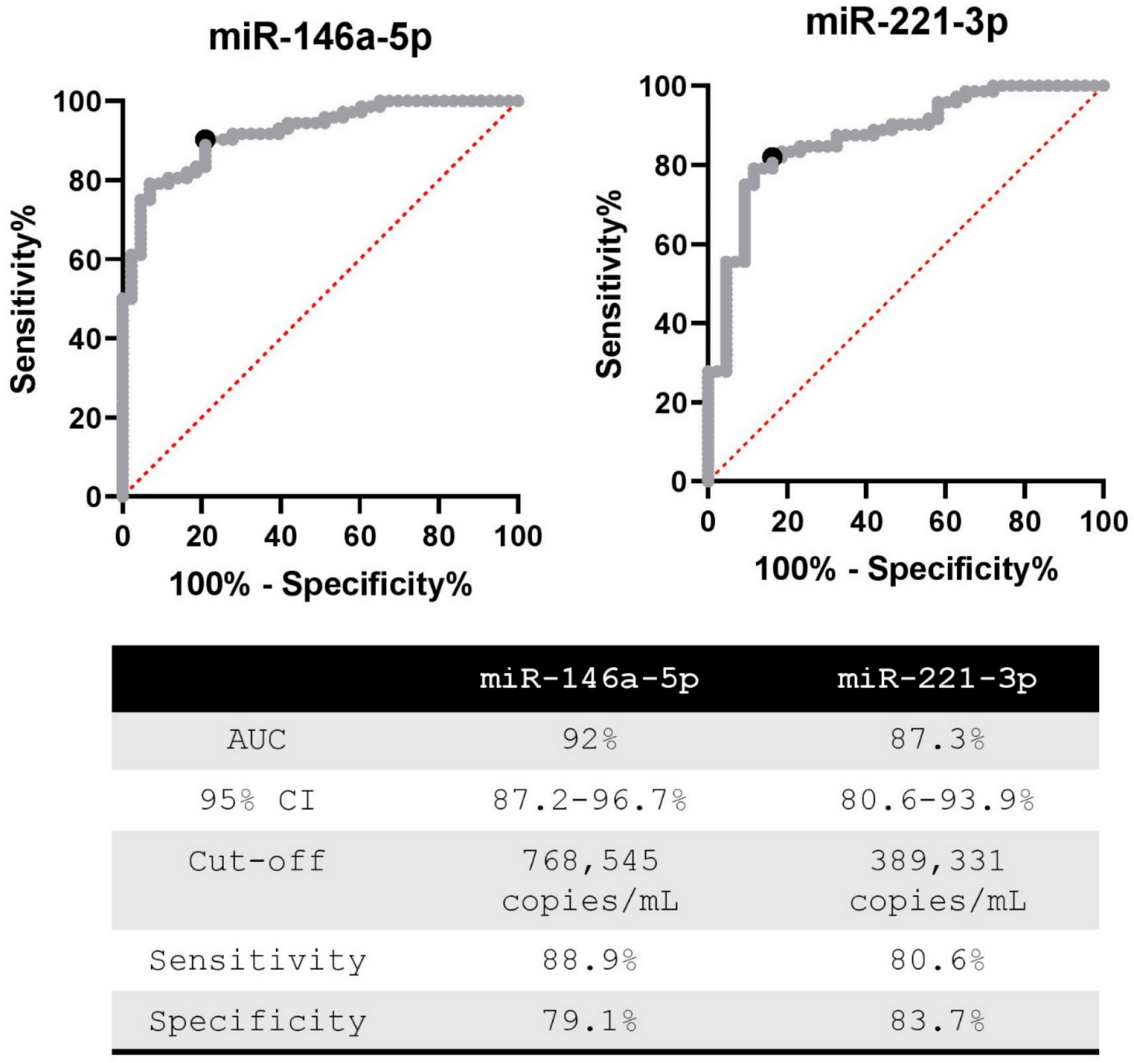
ROC analysis of miRNA levels in the sera of pre-surgery PTC and healthy subjects

ROC analysis of miRNA levels across all samples revealed an area under the curve (AUC) of 89% and 84% for miR-146a-5p and miR-221-3p, respectively. After excluding two HCs with insufficient RNA for repeat measures, the AUC increased to 92% and 87%, for miR-146a-5p and miR-221-3p, respectively (Fig. 1). For miR-146a-5p, a cut-off of 768,545 copies/mL yielded a sensitivity of 88.9% and specificity of 79.1%. For miR-221-3p, a cut-off of 389,331 copies/mL resulted in a sensitivity of 80.6% and specificity of 83.7%. However, the combination of both miRNAs did not enhance the diagnostic performance (AUC 91.1%).

### Evaluation of miR-146a-5p and mir-221-3p as potential PTC markers during the post-surgery surveillance

The clinicopathological features of PTC patients with post-surgery blood samples (*n* = 50) are reported in Table 2.

#### Analysis of absolute miR-146a-5p and mir-221-3p levels in sera samples of patients with excellent response (ER)

All the patients with excellent response (ER, *n* = 14) were confirmed disease-free at their last follow-up visit (median 7.5 years, range 5–10).

**Fig. 2:**
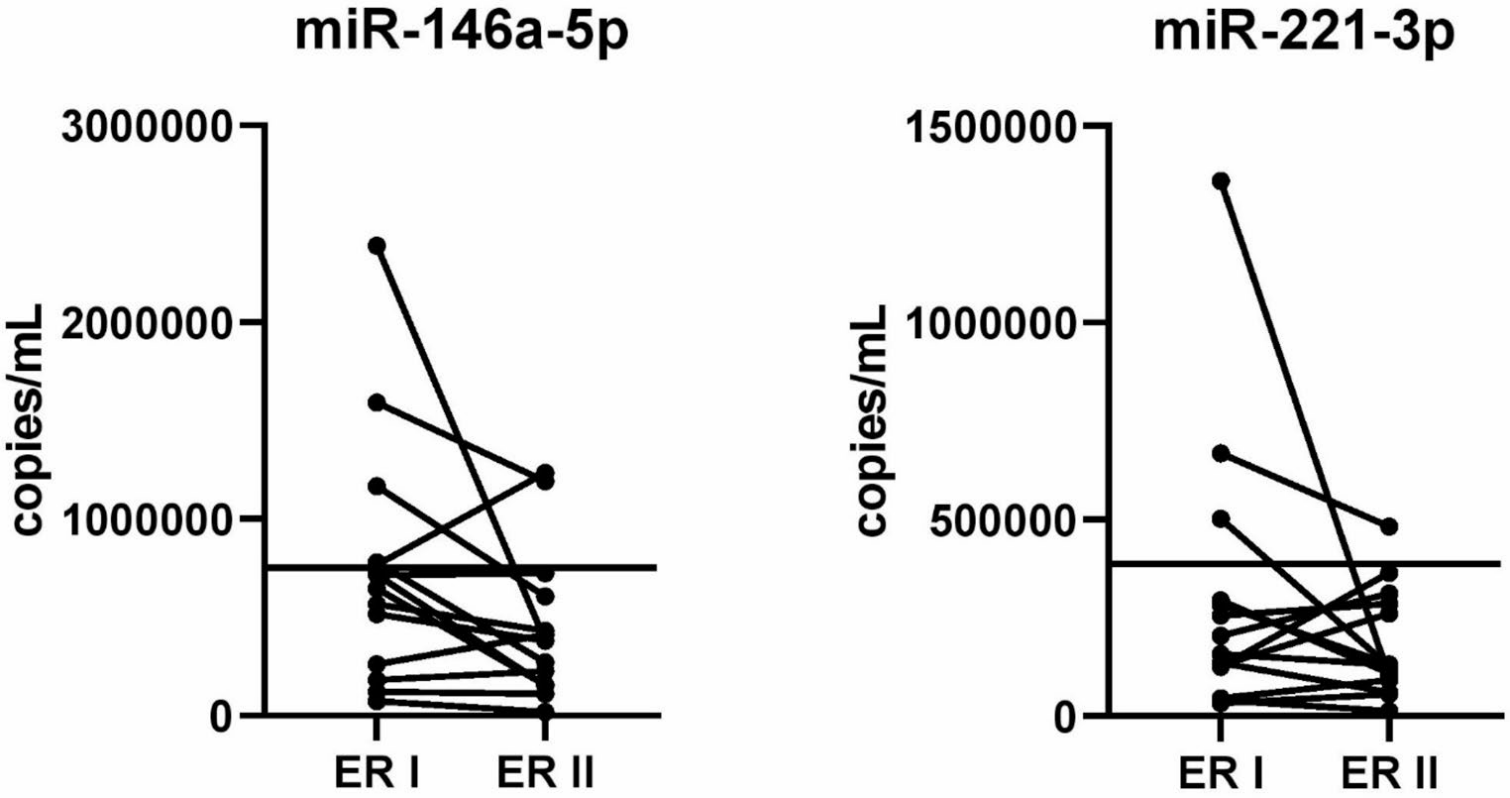
miR levels in the first (ER I) and second (ER II) post-operative serum samples from patients with excellent response (ER). The lines represent the cut-off calculated between pre-surgery PTC and HC sera

Circulating miRNA levels were analyzed at 1–3 months (ER I) and 1–4 years (ER II) post-surgery (Fig. 2).

The mean value of miR-146a-5p was 749,122 and 449,704 copies/mL in the ER I and ER II post-surgery samples, respectively. There was no statistically significant difference between the two groups. Four ER I and two ER II samples showed a value above the previously established cut-off (768,545 copies/mL). In four patients (28.6%), we observed a slight increase in miRNA levels between the first and second blood samples.

The mean value of miR-221-3p was 302,675 and 180,537 copies/mL in ER I and ER II post-surgery samples, respectively. There was no statistically significant difference between the two groups. Levels above the previously established cut-off (389,331 copies/ml) were observed in three ER I and one ER II samples. In five ER patients (35.7%), miRNA levels were slightly increased between the first and second blood samples.

Thus, the trends of miR-146a-5p and miR-221-3p were consistent with the disease status in 71.4% and 64.3% of ER patients, respectively. All patients with increased miR-146a-5p levels also exhibited increased miR-221-3p levels.

#### Analysis of absolute miRNA-146a-5p and mir-221-3p levels in serum samples of patients with structural incomplete response (SIR)

We analyzed the miRNA levels at 1–5 years (SIR I) and 2–16 years (SIR II) after surgery in serum samples of patients with stable (sSIR) (*n* = 6) and progressive disease (pSIR) (*n* = 11). From 4 SED patients, we collected one blood sample during the stable phase and one during disease progression, as confirmed by imaging data. From a fifth patient, we collected two blood samples during the stable phase.

**Fig. 3:**
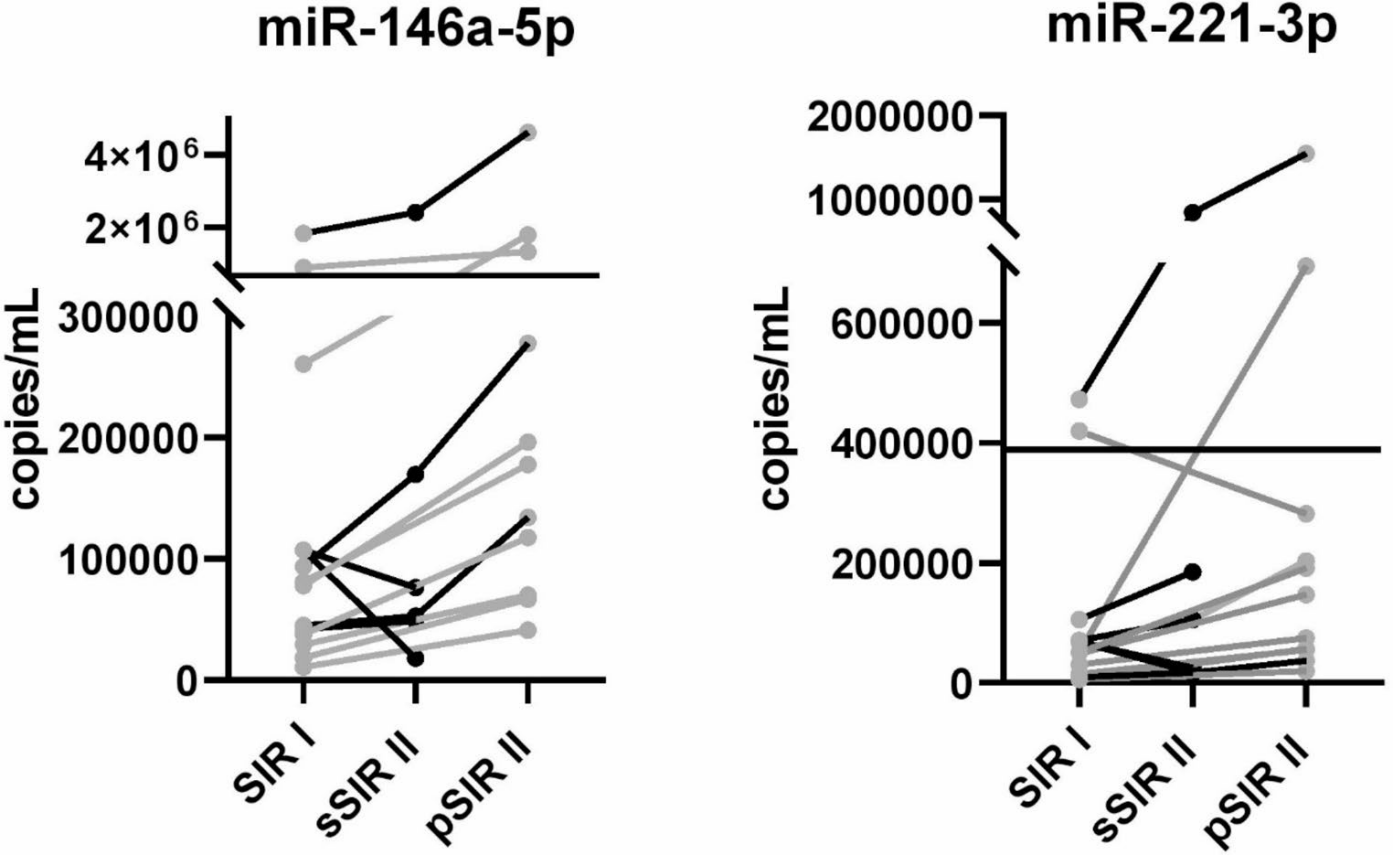
miRNA levels in the first (SIR I) and second (SIR II) post-operative serum samples from patients with structural incomplete response and either progressive (pSIR) or stable (sSIR) disease. The lines represent the cut-off calculated between pre-surgery PTC and HC sera

The mean values of the miR-146a-5p and miR-221-3p were 271,039 and 103,914 copies/mL in the SIR I, 463,420 and 198,712 copies/mL in the sSIR II, and 800,992 and 300,442 copies/mL in the pSIR II post-surgery samples. Both miRNAs didn’t show any statistically significant difference among the three groups (SIR I, sSIR II, pSIR II). Only six (two SIR I, one sSIR II, three pSIR II) and five (two SIR I, one sSIR II, two pSIR II) out of all the 28 analyzed SIR samples showed miR-146a-5p and miR-221-3p values respectively above their previously established cut-offs (768,545 and 389,331 copies/mL, respectively). We observed increased levels of miR-146a-5p in all pSIR II samples (100%) and all but two sSIR II samples (66,7%), and of miR-221-3p in all but one pSIR II samples (90,9%) and all but two sSIR II samples (66,7%) compared with the SIR I samples. (Fig. 3). The trends of miR-146a-5p and miR-221-3p correctly identify patients with a progressive disease in 100% and 90.9%, respectively. Notably, four pSIR and two sSIR patients had TgAbs.

#### Evaluation of miRNA-146a-5p and mir-221-3p fold-change levels as markers of progressive disease

**Fig. 4:**
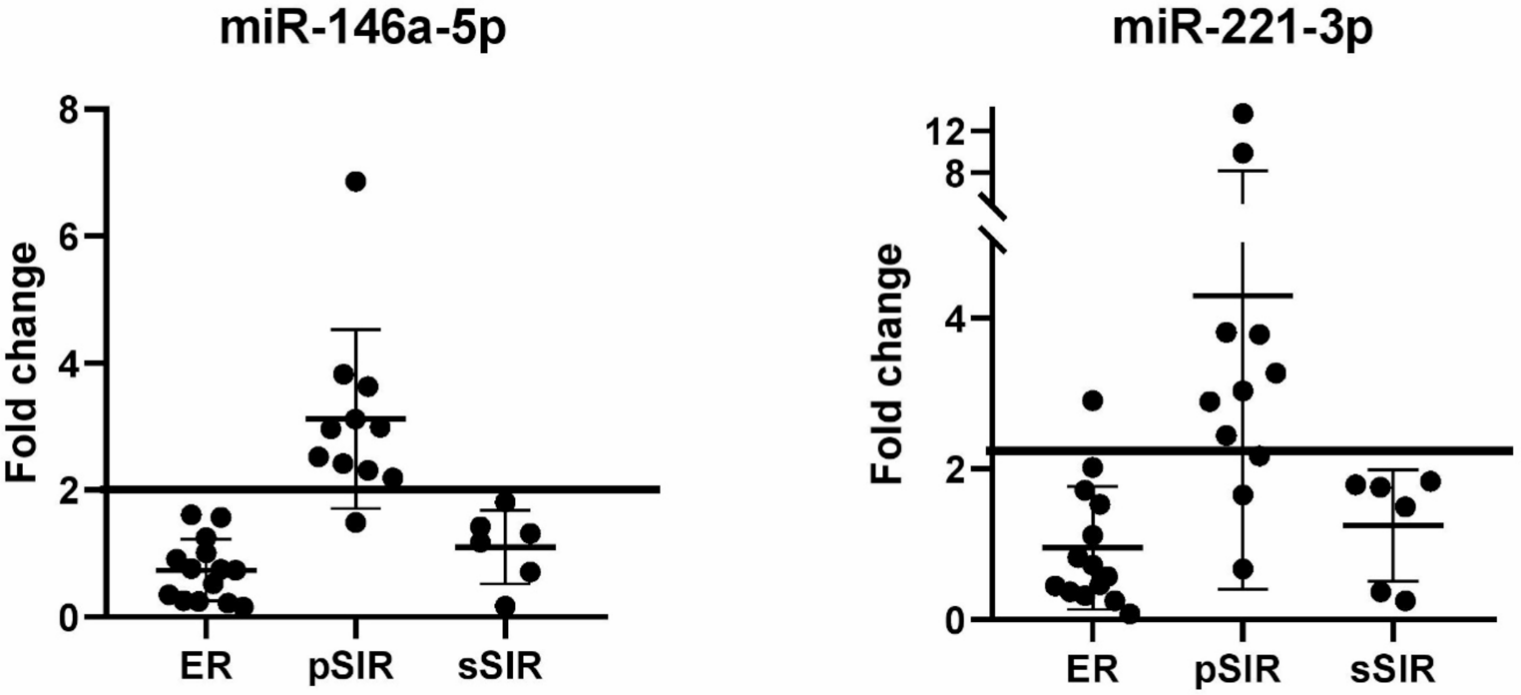
miRNA fold change values between the two post-surgery blood samples from patients with excellent response (ER) and patients with structural incomplete response and progressive (pSIR) or stable (sSIR) disease

Figure 4 shows the fold-change levels of these miRNAs between the two blood samples (II and I) of each ER and SIR patient. The fold-change values of both miRNAs in ER and SIR patients with stable disease (sSIR) were found to be comparable.

**Fig. 5:**
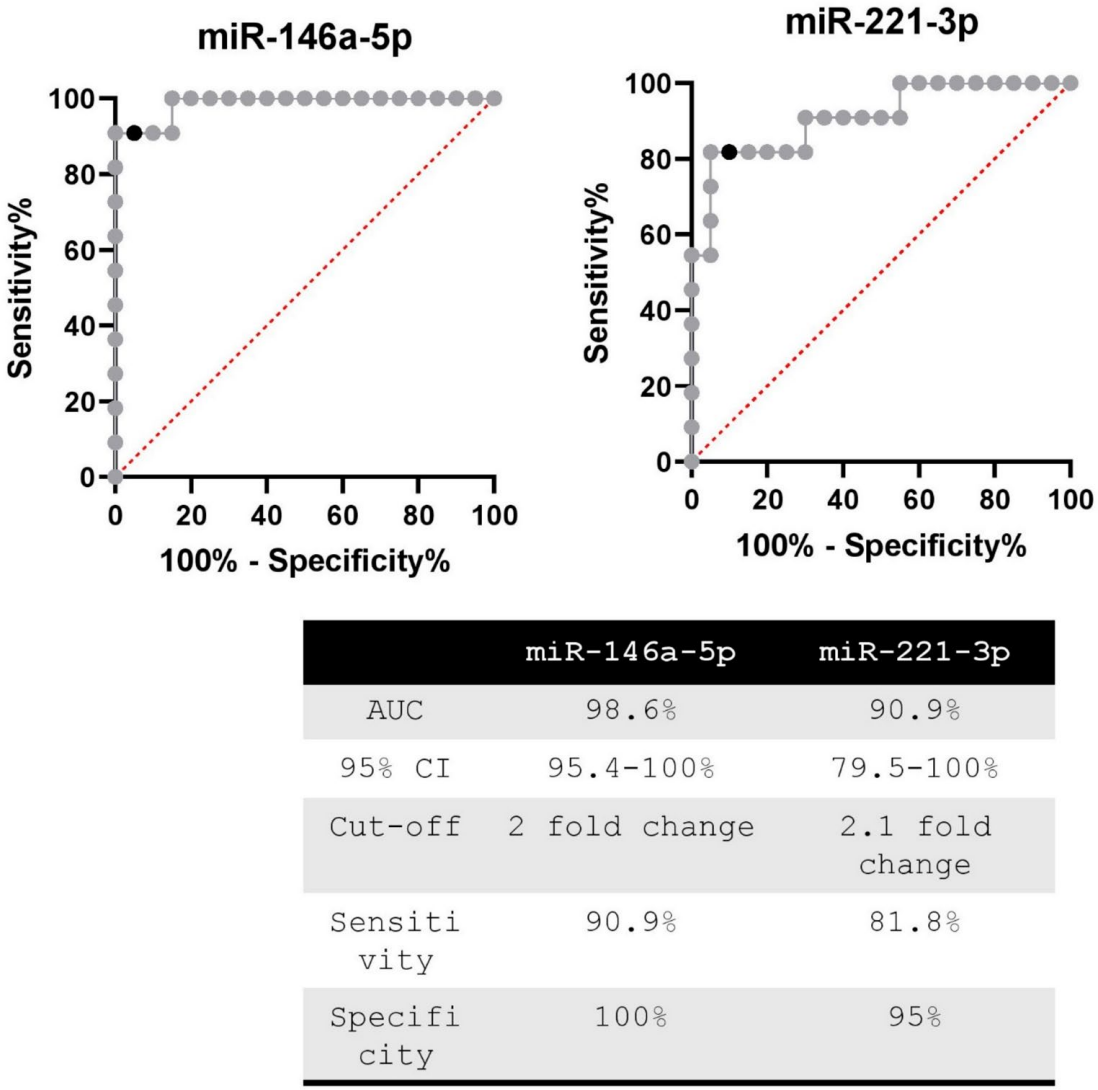
ROC analysis conducted on the miRNA fold changes between the two post-surgery blood samples to distinguish patients with excellent response to surgery (ER) and structural incomplete response with stable disease (sSIR) from those with structural incomplete response and progressive (pSIR) disease

The ROC analysis, as shown in Fig. 5, indicates that a fold change cut-off of 2 for miR-146a-5p can discriminate between patients with a progressive disease and those with a stable or absent disease with a sensitivity and specificity of 91% and 100%, respectively. For miR-221-3p, a fold change cut-off of 2.1 can discriminate between the two groups with a sensitivity and specificity of 82% and 95%, respectively. However, the combination of both miRNAs did not improve the analysis (AUC 95.9%).

#### Analysis of absolute miRNA-146a-5p and mir-221-3p levels in blood samples of patients with indeterminate and biochemical incomplete response (IndR/BIR) and with residual thyroid tissue (Thy Residue)

All patients with IndR/BIR response were confirmed to be disease-free at imaging evaluation at the last follow-up visit (median FU 7 years, range 2–10 years).

**Fig. 6:**
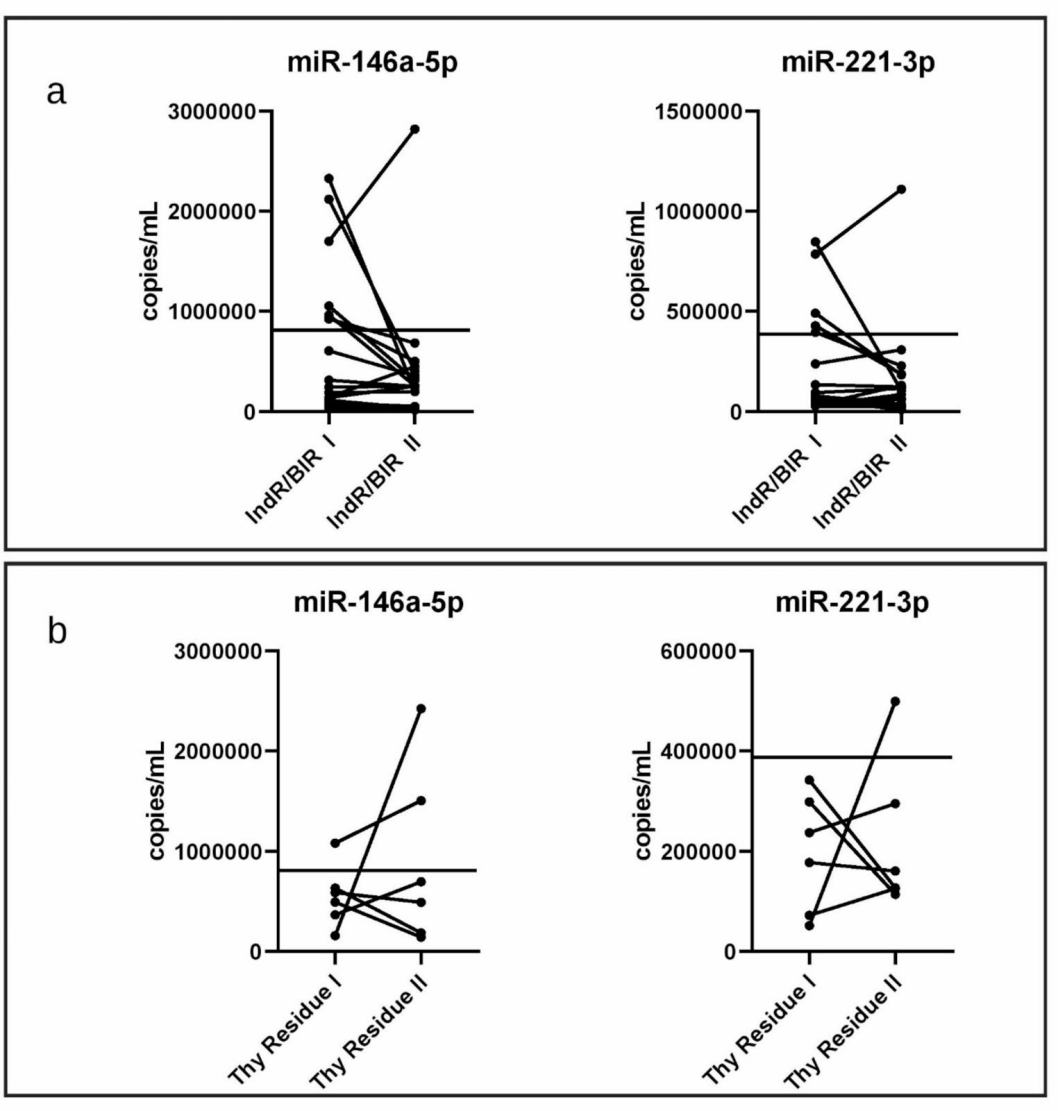
Panel **a**: miR levels in the first (IndR/BIR I) and second (IndR/BIR II) post-operative serum samples from patients with an indeterminate and biochemical incomplete response. Panel **b**: miR levels in the first (Thy Residue I) and second (Thy Residue II) post-operative serum samples from patients with residual thyroid tissue. The lines represent the cut-off calculated between pre-surgery PTC and HC sera

In 12/17 (70.6%) IndR/BIR patients and 3/6 (50%) patients with thyroid residue, we observed a decrease in miR-146a-5p levels between sample I (IndR/BIR I or Thy Residue I) and II (IndR/BIR II or Thy Residue II) (Fig. 6).

When we applied the copies/mL cut-off previously identified, we observed greater values in seven samples I (41.2%) and one sample II (5.9%) from the IndR/BIR group, as well as in one sample I (16.7%) and two samples II (33.3%) from the Thy Residue group.

Decreasing miR-221-3p levels between sample I (IndR/BIR II or Thy Residue II) and II (IndR/BIR I or Thy Residue I) were observed in 10/17 (58.8%) IndR/BIR patients and 3/6 (50%) patients with thyroid residue (Fig. 6). Samples with greater values than the previously identified miR-221-3p copies/mL cut-off were 5 IndR/BIR I (29.4%), 1 IndR/BIR II (5.9%), and 1 Thy Residue II (16.7%).

**Fig. 7:**
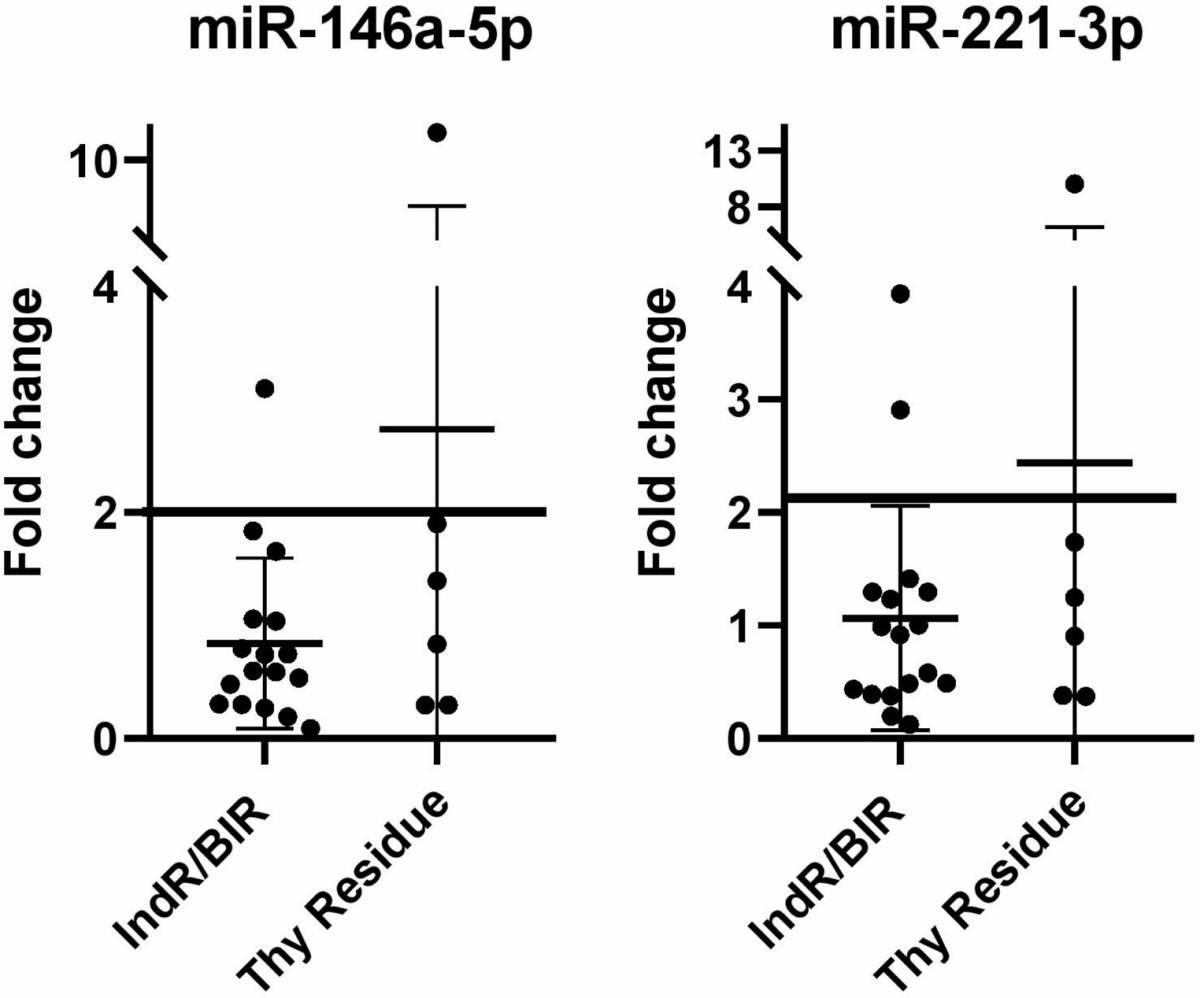
miRNA fold change values between the two post-surgery blood samples from patients with indeterminate and biochemical incomplete response (IndR/BIR) and residual thyroid tissue (Thy Residue). The lines represent the cut-off to distinguish patients with excellent response to surgery (ER) and structural incomplete response with stable disease (sSIR) from those with structural incomplete response and progressive (pSIR) disease

When examining the fold-change values, we found that two (8.7%) and three (13%) patients with IndR/BIR response or residual thyroid tissue had values above the cut-off for miRNA-146a-5p and miRNA-221-3p, respectively (Fig. 7).

Therefore, the trends of miR-146a-5p and miR-221-3p were consistent with the disease status in 70.6% and 58.8% of IndR/BIR, respectively, and 50% of Thy Residue patients. Using the fold-change cut-off, we reached the 94.1% (16/17) and 88.2% (15/17) for IndR/BIR, respectively, and 83.3% (5/6) for Thy Residue patients. Interestingly, both patients with high miR-146a-5p fold-change also had high miR-221-3p fold-change.

## Discussion

Liquid biopsy, a non-invasive, rapid, and cost-effective method, provides access to tumor-derived material, including microRNAs (miRNAs). However, results from circulating miRNA analysis in cancer are often discordant and lack cross-study validation. This inconsistency can be attributed to the pre-analytical, analytical, and biological variabilities. Biological variability encompasses both intra-individual factors (e.g., time of the day, diet, sex, age, alcohol consumption) and inter-individual factors such as the presence of conditions that modulate miRNA levels (e.g., inflammatory and immune conditions) [27].

Our previous pilot study identified serum miRNA-146a-5p and miRNA-221-3p as potential biomarkers for PTC diagnosis and for detecting recurrent/persistent disease during patients’ follow-up [23]. In the present study, we further investigated the clinical utility of the miRNA-146a-5p and miRNA-221-3p on larger cohorts of PTC patients and healthy controls (HCs) using a more sensitive technology, digital PCR. Indeed, we confirmed the role of both miRNAs as diagnostic biomarkers for papillary thyroid cancer and their usefulness in monitoring cancer progression during patient follow-up, even when thyroglobulin levels are not informative.

Zhang Y. and colleagues previously reported the diagnostic role of serum miRNA-221-3p, finding elevated levels in PTC compared to HCs sera, with an AUC of 91.2%. They also found an association between the miRNA-221-3p levels and specific clinical features (e.g., tumor location, extrathyroidal invasion, metastatic lymph node, and TNM stage) [21]. While we confirmed the high diagnostic potential of miRNA-221-3p reported in our previous paper with a slightly lower but still excellent AUC (87.3% vs. 93%) [23], we didn’t find any association between its levels and the clinical features.

Several studies have reported the diagnostic role of serum miR-146a-5p being downregulated in PTCs vs. HCs [28, 29]. In contrast, our study confirmed the upregulation of miR-146a-5p in PTCs’ sera and the high AUC value (92% vs. 90%) as reported in our previous paper [23].

Thyroglobulin (Tg) is a cost-effective and essential biomarker for monitoring differentiated thyroid cancer (DTC) follow-up, especially for low- and intermediate-risk patients with excellent treatment response [11], as per thyroid cancer guidelines [12]. However, its effectiveness is limited by anti-thyroglobulin antibodies (TgAb) in 20–25% of patients, residual thyroid tissue, and thyrotropin levels [13, 14]. These conditions highlight an area of unmet need. Naturally, a key question arises: Can liquid biopsy, particularly microRNAs, serve as a viable alternative to Tg measurement in these scenarios?

Only some studies have attempted to explore the role of serum miRNAs in PTC patients during follow-up, underscoring the challenges inherent to data analysis and interpretation.

Campenni et al. examined the levels of several miRNAs (miR-221, miR-222, miR-375, miR-155, and miR-146b) in the sera of PTC patients with positive TgAbs at 6 and 12 months after the initial treatments (surgery and radioiodine ablation) compared to baseline levels (just before the radioiodine treatment). They observed a reduction of ≤ 50% exclusively in the group of patients with excellent responses but not in those with incomplete structural or indeterminate/ biochemical incomplete responses. However, the study reported only the mean miRNA levels for each patient group rather than individual patient trends [10]. Zhang et al. investigated the expression levels of miR-222, miR-221, and miR-146b in the serum of PTC patients at recurrence and found elevated levels of all miRNAs compared to non-recurrent patients [21]. Conversely, Miljus et al. found no known miRNA as helpful markers of recurrence by using two different approaches (small RNA sequencing and two qPCR platforms) [20].

Our study focused on two aspects: the potential for detecting disease progression and for providing information on disease status when thyroglobulin is unreliable (i.e., in the presence of AbTg, residual thyroid tissue, and indeterminate Tg values).

Our findings indicate that: (i) the absolute level cut-off distinguishing pre-surgery PTC from HC sera doesn’t apply to post-surgery samples for detecting disease presence and progression. This discrepancy could be attributed to varying miRNA levels in subjects with a thyroid gland (whether diseased or healthy) and in those who are thyroidectomized, as well as to inter-individual variability; (ii) the reliability of analyzing the trend of absolute miRNA levels in patients during follow-up is questionable. Indeed, we found a slight increase in miRNA levels in the second post-surgery sample compared to the first one in 28,6% and 42.9% of patients with excellent response (for miRNA-146a-5p and miRNA-221-3p, respectively). This could be explained by the high intra-individual variability causing fluctuations in miRNA levels over time. However, using the fold change values relative to the first available post-surgery sample appears more trustworthy and helps overcome such variability. We found that a doubling or more of miRNA levels indicates disease progression. This approach enabled us to identify progressive disease with a sensitivity and specificity of 92% and 100%, and 75% and 95% for miRNA-146a-5p and miRNA-221-3p, respectively.

In post-surgery samples of patients with indeterminate/biochemical incomplete response and with thyroid remnants, by applying the fold change cut-off, we were able to correctly exclude the disease progression, following the RECIST criteria 1.1, in all but 2 and 3 patients (91,3% and 87%) for miRNA-146a-5p and miRNA-221-3p respectively, according to the disease status at the last follow-up. This suggests that the presence of TgAbs or remnant thyroid doesn’t significantly influence the miRNA fold changes.

Overall, miRNA-146a-5p seems to be more useful than miRNA-221-3p in both PTC diagnosis and disease monitoring during patient follow-up. Three out of fifty patients with post-surgery blood samples (6%) were incorrectly classified by miRNA-146a-5p. Interestingly, these patients also showed the same trend for miRNA-221-3p, suggesting the presence of miRNA-modulating conditions (e.g., inflammatory, and immune). However, we couldn’t find any recorded clinical information that may explain the miRNAs’ trend.

Some limitations of the current research include the variable timing of blood collection and the relatively limited number of patients whose post-surgery blood samples were evaluated. These limitations are partly attributable to the retrospective aspect of the study. Moreover, although we avoided clearly hemolyzed samples, we didn’t perform a hemolysis evaluation. This omission may have introduced some bias. On the other hand, we aimed to ascertain whether we could analyze blood samples typically processed in a routine laboratory. Our study’s design enabled us to investigate the role of miRNAs in identifying the disease progression but not the presence of minimal residual disease after the first treatment.

Finally, several aspects still require clarification. Future investigations should determine if circulating miRNAs can be used for disease monitoring during the follow-up when other treatments (e.g., RAI) are administered, establishing the more appropriate timing for blood sample collection. Another critical consideration is whether miRNAs can identify disease progression earlier than the existing biochemical or radiological evaluations.

## Electronic supplementary material

Below is the link to the electronic supplementary material.

Supplementary Material 1

## Data Availability

The data that support the findings of this study are available from the corresponding author upon reasonable request.

## Abbreviations

PTC: Papillary thyroid carcinoma
Pre-PTC: Pre-surgery PTC sample
ER: Excellent response
IndR/BIR: Indeterminate and biochemical incomplete response
SIR: Structural incomplete response
sSIR: Structural incomplete response with stable disease
pSIR: Structural incomplete response with progressive disease
HC: Healthy controls
dPCR: Digital PCR
Tg: thyroglobulin
TgAb: Anti-Tg antibodies
US: Ultrasound
Thy Residue: Residual thyroid tissues
ROC: Receiver-operating characteristic
