# Supplementary material for: Serum microRNA-146a-5p and microRNA-221-3p as potential clinical biomarkers for papillary thyroid carcinoma
Source: J Endocrinol Invest. 2024 Sep 19;48(3):619–31. doi: 10.1007/s40618-024-02467-3 (PMC11876262; doi:10.1007/s40618-024-02467-3)
Supplement: Supplementary file 1 — Supplementary Material 1 [file 40618_2024_2467_MOESM1_ESM.docx]

| **Table S1. Metabolic features of PTCs with pre-surgery blood samples** | | | |
| --- | --- | --- | --- |
|  | **PTC Pre-surgery (n=72)** | **miR-146a-5p p-value^‡^** | **miR-221-3p p-value^‡^** |
| **BMI** | **N (%)** | ns | ns |
| 18.50-24.99 | 27 (37.5) |  |  |
| 25-29.99 | 24 (33.3) |  |  |
| 30-34.99 | 14 (19.4) |  |  |
| NA | 7 (9.8) |  |  |
| **Metabolic dysregulation** | **N (%)** | ns° | ns° |
| Yes | 15 (20.8) |  |  |
| Insulin resistance | 3 (20) |  |  |
| Diabetes* | 5 (33.3) |  |  |
| Dyslipidemia | 12 (80) |  |  |
| No | 56 (77.8) |  |  |
| NA | 1 (1.4) |  |  |
|  |  |  |  |
| * All diabetic patients had dyslipidemia |  |  |  |
| ^‡^Kruskal-Wallis test with Dunn's multiple comparisons | |  |  |
| ° Comparison between each dysregulated and normal metabolic status | | | |
